# A Novel Polysaccharide Isolated From Fresh Longan (*Dimocarpus longan* Lour.) Activates Macrophage *via* TLR2/4-Mediated PI3/AKT and MyD88/TRAF6 Pathways

**DOI:** 10.3389/fphar.2021.786127

**Published:** 2021-12-21

**Authors:** Haibo Lan, Wu Li, Jucai Xu, Yuzhe Yang, Zhaolun Tan, Ruili Yang

**Affiliations:** ^1^ School of Biotechnology and Health Sciences, Wuyi University, Jiangmen, China; ^2^ College of Food Science, South China Agricultural University, Guangzhou, China; ^3^ School of Biotechnology, Sichuan University of Science & Engineering, Yibin, China

**Keywords:** biomacromolecules, polysaccharide, longan, toll-like receptors, PI3K/Akt pathway

## Abstract

A novel immunomodulatory polysaccharide (LP4) with a molecular weight 6.31 × 10^4^ g/mol was purified from fresh longan pulp. It was composed of mannose, glucose, glucuronic acid, galactose, xylose, arabinose, galacturonic acid, fucose, and rhamnose in a molar percentage of 36:31:10:7:4:4:3:2:2, and mainly linked by (1→6)-*β*-Man, (1→4)-*β*-Glc and (1→6)-*α*-Glc. LP4 can obviously enhance the phagocytosis of macrophages and promote the proliferation of lymphocytes. After treating macrophages with LP4 (12.5–50 μg/ml), the production of IL-1β and TNF-α was significantly increased. These increases of cytokines were suppressed when the TLR2/TLR4 receptors were inhibited by anti-TLR2 and/or anti-TLR4 antibodies. Moreover, the mRNA expression of INOS, AKT, PI3K, TRAF6 and MyD88 was significantly suppressed by TLR2/TLR4 antibodies. These results indicated that LP4 induced macrophage activation mainly *via* the TLR2 and TLR4-induced PI3K/AKT and MyD88/TRAF6 pathways.

## Introduction

Polysaccharides, a kind of bio-macromolecule which are widely distributed in animals, plants and microorganisms, were extensively involved in cell proliferation, differentiation and signal transduction (Xie et al., 2016). A number of pharmacological activities of natural polysaccharides have been reported, such as antiviral, antitumor, and immunomodulatory activities (He et al., 2020; López-Legarda et al., 2020). Recently, several natural polysaccharides such as *Ganoderma* polysaccharide, *Astragalus* polysaccharide and *Lentinan* polysaccharide are used for clinical treatment of cancer in combination with chemotherapy (Yu et al., 2018; Lin and Sun, 2019). Although polysaccharides from various natural sources have been displayed significant biological activities, the application of many natural polysaccharides in medicine and functional foods are limited due to the unclear of composition and structure (Wang et al., 2018b).

Longan (*Dimocarpus longan* Lour.) is a well-known ‘Medicinal and Edible’ fruit in China and Southeastern Asia. Longan was used to improve the immunity and treat disease such as palpitation, amnesia, neurasthenia, and relieving fatigue (Zhang et al., 2017). Recent researches showed that longan polysaccharides have a various bioactivities, including antioxidative (Yang et al., 2011), antitumor (Meng et al., 2014) immunoregulatory activities (Yi et al., 2015; Zhang et al., 2017; Rong et al., 2019), regulating intestinal flora and intestinal metabolites (Zhang et al., 2017; Bai et al., 2020).

In previous studies, several active polysaccharides have been isolated from longan. However, there are differences in the structural characteristics of longan polysaccharides in these previous reports. An active polysaccharide isolated from longan composed of →6)-Glc-(1→, →5)-Ara-(1→, →4)-Man-(1→ and →6)-Gal-(1→ can increase the inducible nitric oxide synthase activity, TNF-α and IL-6 secretion of macrophages (Yi et al., 2015). A longan polysaccharide obtained by Meng et al. (2014), which composed of glucose, arabinose, galactose and galacturonic acid, stimulated the production of IFN-γ and increased the phagocytic ability of macrophages. Zhu et al. (2013) obtained a homogeneous active polysaccharide from longan composed of (1→6)-*α*-glucan. In early research (Rong et al., 2019), we purified an active polysaccharide (LPD2) from longan, which composed of (1→4)-*β*-Glc with substituted by acetyl, and (1→6)-*β*-Man, and induced macrophage activation *via* the TLR2-and TLR4-mediated MyD88/IRAK4-TRAF6 pathways. The differences in the structure of polysaccharides in these reports may be due to different extraction methods or drying processes (Gan et al., 2021). However, there may be multiple polysaccharides in longan that contribute to the bioactivity of longan. The study on the composition and structure of longan active polysaccharides may help to clarify their principal active components and furtherly apply in medicine.

In this study, a novel polysaccharide (LP4) was purified from fresh longan. The structure of LP4 was characterized and its immune regulation activity was evaluated. Present study will contribute to reveal the active polysaccharide composition and potential mechanism of immunoregulation of longan polysaccharide.

## Materials and Methods

### Materials

Longan fruit (cv. Chuliang) was obtained from a local orchard (Gaozhou, Guangdong province, China) which was harvested at maturity stage in 2016. The RAW264.7 cells were purchased from Cell Bank of Chinese Academic of Science (Shanghai, China). DMEM medium, RPMI-1640 medium, fetal bovine serum, Anti-TLR2 (6C2) and Anti-TLR4 (MTS510) antibodies were purchased from Thermo Fisher Scientific (Waltham, MA, United States). Polymyxin B, neutral red and lipopolysaccharide (LPS) were purchased from Sigma-Aldrich (Shanghai, China).

### Extraction and Purification of Polysaccharides

The longan fruit were peeled, stoned and homogenized using a homogenizer (30 s × 2, C91T, Joyang Co., Ltd., Jinan, China). The homogenates were extracted with distilled water at 80°C for 3 h. The extracts were filtrated with gauze (74 μm) and then centrifuged (3,000 g/min) for 20 min. The liquid supernatant was concentrated to 1/10 the original volume by rotary evaporation at 55°C and then discarded protein and pigment by D301R resin (Tianjin Bohong Resin Co., Ltd., Tianjin, China) using our previous method (Yi et al., 2012). After centrifuging, the filtrates were dialyzed (8,000–14,000 Da) 3 days in distilled water to remove small molecule compounds. A crude longan polysaccharide was collected *via* freeze-drying. The crude polysaccharide (10 mg/ml) was fractionated with a DEAE-fast flow column (2.0 cm × 30 cm), which was eluted with different concentrations of sodium chloride (0, 0.02, 0.05, 0.1 mol/L). The eluate (3.0 ml/tube) was monitored *via* the phenol-sulfuric acid method at 490 nm to find out the polysaccharide components (Masuko et al., 2005). The eluting profiles is shown in Supplementary Figure S1. The eluted fractions (eluted by 0.05 mol/L NaCl) were further purified on a liquid chromatography system (Agilent 1200, refractive index detector, Santa Clara, CA, United States) with TSKgel-G3000PW_XL_ in series with TSKgel-G4000PW_XL_ (7.8 mm I. D × 30 cm) (TOSOH, Tokyo, Japan) columns. The sample was eluted with 0.6 ml/min water at 55°C. The main fraction peaks (LP4) were collected in the light of the retention time and peaks. This samples were collected and lyophilized.

### Structural Analysis of LP4

#### SEC-MALLS

The molecular features of LP4 were confirmed with high-performance size exclusion chromatography (Waters 2695, Milford, MA, United States) coupled with a multi-angle laser light scattering detector (DAWN HELEOS-II, Wyatt Technology, Santa Barbara, CA, United States) and refractive index detector (Optilab DSP, Wyatt Technology, Santa Barbara, CA, United States) (SEC–MALLS–RID). LP4 was analyzed on a Ultrahydrogel 2000 (Waters, Milford, MA, United States) column and eluted with 0.1 mol/L NaNO_3_ (0.5 ml/min). The column temperature was kept at 35°C. The data were analyzed with ASTRA 5.3.4.20 software.

#### Monosaccharide Composition

The monosaccharide composition was analyzed according to previously published method (Wang et al., 2017). Briefly, the sample (10.0 mg) was completely hydrolyzed in 2 ml trifluoroacetic acid (2.0 mol/L) at 121°C for 2 h. After adjusting pH = 7.0, NaCO_3_ (0.5 mol/L, 80 µL) was added and incubated for 45 min at 30°C. Thereafter 50.0 mg NaBH_4_ (4%, w/v) was added and maintained at room temperature for 2 h. The remaining reagent was neutralized by 25% (v/v) glacial acetic acid. Cation exchange column was used to remove Na^+^ and boric acid was washed off with methyl alcohol. After that, the residue was place in a vacuum drying oven for 2 h at 85°C to convert the uronic acid salt as lactone. In the end, pyridine (1 ml) and normal propyl amine (1 ml) were added for 30 min at 55°C. After drying, the residue was dissolved in 1 ml pyridine and 1 ml acetic anhydride at 100°C for 1 h. After evaporation drying the sample was dissolved in 2 ml CH_2_Cl_2_ and filtered (0.45 μm pore size), then was analyzed using GC. The injection temperature was 180°C and kept for 3 min, then went up to 210°C by 0.3°C/min and finally increased to 240°C and held for 30 min. The carrier gas was N_2_ with the rate of 0.88 ml/min. The split ratio was 19:1 and the pressure was 110 kPa.

#### GC-MS

The methylation analysis of LP4 was conducted according to the previously published method (Needs and Selvendran, 1993; Rong et al., 2019). Briefly, 8.0 mg samples and 200 mg NaOH were dissolved in 5.0 ml anhydrous dimethyl sulfoxide and stirred for 4 h. Then 1.5 ml methyl iodide were added and light-tight reaction for 2 h. This chemical reaction was terminated by adding 4.0 ml of distilled water and extracted with 3.0 ml CHCl_3_. The samples was dried with a rotary evaporation and then hydrolyzed by trifluoroacetic acid and acetylation with acetic anhydride and pyridine. At last, the samples were extracted with CHCl_3_ and analyzed by GC-MS (Agilent 7890–5977, Agilent, Santa Clara, CA, United States) equipped with an DB-225 column (J&W Scientific, Folsom, CA, United States). The operation conditions were set as follows: the initial column temperature was 55°C for 0.75 min, increased to 140°C at 45°C/min. After keeping 1 min, the temperature was raised to 218°C with a rate of 2.5°C/min and keeping for 37 min. Linear velocity of the carrier gas (H_2_) was set at 50 cm/s at 218°C. The mass spectrometer was operated in the electron impact mode (EI) at 70 eV scanning the range 50–700 m/z, in a full scan acquisition mode.

#### FT-IR Analysis

The infrared spectral characteristics of LP4 was characterized by infrared spectrophotometer (TENSOR27, Bruker, Karlsruhe, Germany). LP4 (1.0 mg) was ground with KBr powder before pressed into pellets, then measured by IR spectral in the range of 4,000–400 cm^−1^.

#### NMR Analysis

LP4 (20.0 mg) was completely dissolved in 0.8 ml D_2_O and then transferred into NMR-tube. ^1^H and ^13^C NMR and 2D (COSY and HSQC) spectra were recorded using a 400 MHz spectrometer (Bruker, Karlsruhe, German) operating at 30°C.

### Evaluation of the Immunoregulation Activities of LP4

#### Phagocytosis Assay

Macrophages phagocytic capacity was referred to a previously method (Weeks et al., 1987; Long et al., 2005). Briefly, RAW 264.7 cells (2×10^5^ cells/mL) were plated into a 96-well plate incubated for 3 h. Then LP4 were treated with 1,000 units/mL polymyxin B (PMB) for 60 min to exclude the effect of lipopolysaccharide (LPS). After LP4 (final concentration: 0, 12.5, 25 and 50 μg/ml) was added for 24 h, the phagocytosis was assessed with 100 μL neutral red (0.1%, w/v) incubated for 4 h. Then, the plate was washed with medium, and a 200 μL cell lysate (1:1 acetic acid to ethanol (v/v) was added and incubated for 1 h. The absorbance at 570 nm was measured with a microplate reader. The phagocytic index was calculated as the relative increase of absorbance value of each sample in relation to the control.

#### TNF-α and IL-1β Assay

The RAW264.7 cells (2 × 10^5^ cells/mL) were seeded into 96-well plates and cultured in DMEM medium at 37°C under 5% CO_2_. After incubation for 4 h, the plates were washed twice by DMEM medium and 200 μL medium with LP4 (0, 12.5, 25 and 50 μg/ml) was added to the wells, followed by incubation for 24 h. The cells cultured in DMEM without LP4 were used as the control group. The levels of TNF-α and IL-1β were measured using the ELISA kits (R&D systems, Minneapolis, MN, United States), respectively. The level was normalized and converted to a value equal to the relative cell density of that control group.

#### Antibody Experiments

Anti-TLR2, anti-TLR4 and anti-TLR2/4 (anti-TLR2 plus anti-TLR4) were used to make sure the effect of TLR2 and TLR4 receptor in the LP4-induced macrophage activation. The cells were inoculated in 96-well plates and then treated with anti-TLR2, anti-TLR4 and anti-TLR2/4 (0.1 μg/ml) for 30 min. Then LP4 (50 μg/ml) was added for 24 h. The supernatant was collected for analyzing the production of TNF-α and IL-1β.

#### Proliferation Assay of Splenic Lymphocytes

The splenic lymphocytes were isolated according to the previous method (Jiang et al., 2014). The cells were inoculated into a 96-well plate (1×10^6^ cells/mL) and cultured with 100 μL/well RPMI-1640 medium. Then LP4 (12.5 μg/ml ~ 50 μg/ml) with or without LPS (10.0 μg/ml) was added for 48 h. Subsequently, 50 μL MTT (5 mg/ml) was added and incubated for another 4 h. Finally, the 100 µL of acidified isopropyl alcohol was added. The optical density was measured at 570 nm with a microplate reader. The proliferation index of splenic lymphocytes was expressed as the percentage of the control group.

#### Real-Time PCR Analysis

LP4 (25 μg/ml) with or without anti-TLR2 and anti-TLR2/4 antibodies was added in RAW 264.7 cells. After incubated 4 h, the total RNA was extracted from cells and cDNA was synthesized from 1 µg of total RNA using a Reverse Transcription System kit (Thermo Fisher Scientific, Shanghai, China). The expression of INOS, PI3K, AKT, MyD88, TRAF6 and TRAF6 genes was assessed by real-time RT-PCR. Beta-actin was used as an internal control. The relative changes in expression of the target gene were derived using the CT (2^−ΔΔCT^) method. The primers sequences used are presented in Supplementary Table S1.

### Statistical Analysis

The Data was expressed as an average of the replicates ±standard deviation. SPSS statistic version 22.0 (SPSS. Inc., Chicago, IL) was used to perform statistical analysis of experimental results. A One-way analysis of variance (ANOVA) was used to evaluate the differences between the control and treatment groups. A *p* value of <0.05 was considered as statistically significant.

## Results

### Molecular Parameters

**FIGURE 1:**
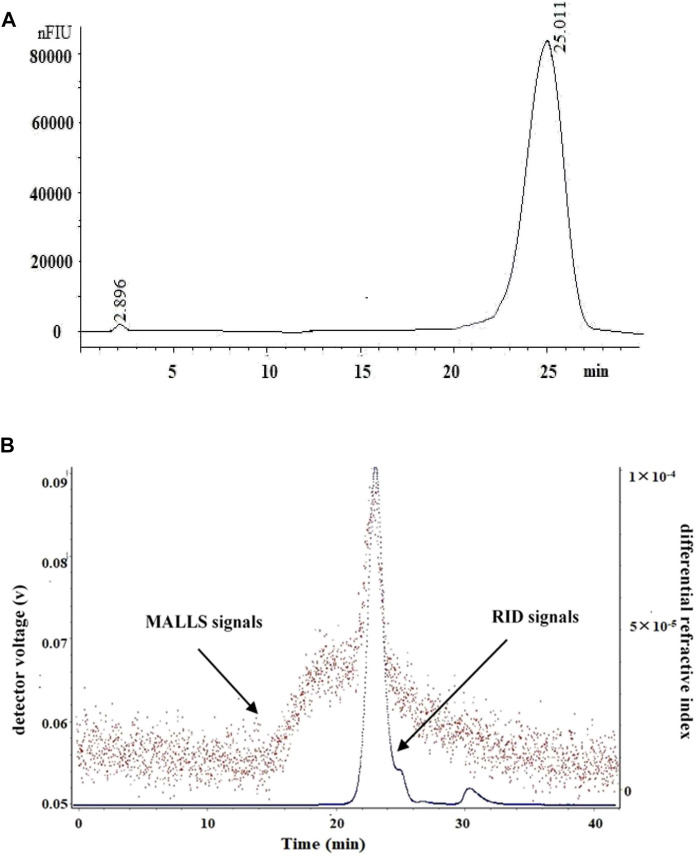
The HPLC and SEC-LLS analysis of LP4 purified from longan pulp. **(A)** HPLC spectrum of LP4; **(B)** SEC-LLS spectrum of LP4. The SEC-LLS of LP4 with laser light scattering and differential refractive index detector in 0.1 mol/L NaNO_3_ at 25°C.

The high-performance liquid chromatography profiles of LP4 showed a single and symmetrical peak at 25.01 min (Figure 1A). Similarly, the result of SEC-MALLS (Figure 1B) showed a major peak at 23.26 min matched with RID signals.

The macromolecular characteristics parameters of LP4 are shown in Table 1. The Mw of LP4 was calculated as 6.31 × 10^4^ (g/mol) and the Mn, Mz and Mp were 3.58 × 10^4^ (g/mol), 2.25 × 10^5^ (g/mol), and 2.87 × 10^4^ (g/mol), respectively. The radius moments of LP4 were 56.32 nm (Rn), 70.13 nm (Rw) and 100.65 nm (Rz).

**TABLE 1:** The molecular weight and size of LP4.

Molar mass moments (g/mol)				Rms radius moments (nm)		
Mn	Mw	Mz	Mp	Rn	Rw	Rz
3.58 × 10^4^	6.31 × 10^4^	2.25 × 10^5^	2.87 × 10^4^	56.32	70.13	100.65
Mw/Mn = 1.76		Mz/Mn = 6.29				

### Monosaccharide Composition Analysis

The monosaccharide composition analysis by GC chromatogram (Supplementary Figure S2) showed that LP4 was composed of mannose, glucose, glucuronic acid, galactose, xylose, arabinose, galacturonic acid, fucose, and rhamnose in a molar percentage of 36:31:10:7:4:4:3:2:2. The monosaccharide composition results indicated that LP4 was a hetero polysaccharide with high proportion of mannose (36.5 mol%) and glucose (31.5 mol%).

### Methylation Analysis

To elucidate the glycosidic bonds configuration of LP4, methylation analysis was carried out. The results of GC/MS are summarized in Table 2. LP4 was mainly composed of (1→6)-man (41.41 mol%), (1→4)-Glc (15.27 mol%) and (1→6)-Glc (15.95 mol%). Meanwhile, low percentage of (1→4)-Gla (6.97 mol%) and (1→4)-Xyl (4.36 mol%) was detected in LP4.

**TABLE 2:** The relative molar percentage of glycosidic linkages in LP4.

Composition	Glycosidic linkages	Relative molar percentage (%)
Mannose	→6)-Man-(1→	41.41
Glucose	→6)-Glc-(1→	15.95 15.27
	→4)-Glc-(1→	
	Glc-(1→	5.21
Galactose	→4)-Gal-(1→	6.97 3.21
	Gal-(1→	
Xylose	→4)-Xyl-(1→	4.36
Arabinose	→5)-Ara-(1→	2.10 1.72
	→3)-Ara-(1→	
Fucose	→4)-Fuc-(1→	2.17
Rhamnose	→2)-Rha-(1→	1.62

### FT-IR Analysis

**FIGURE 2:**
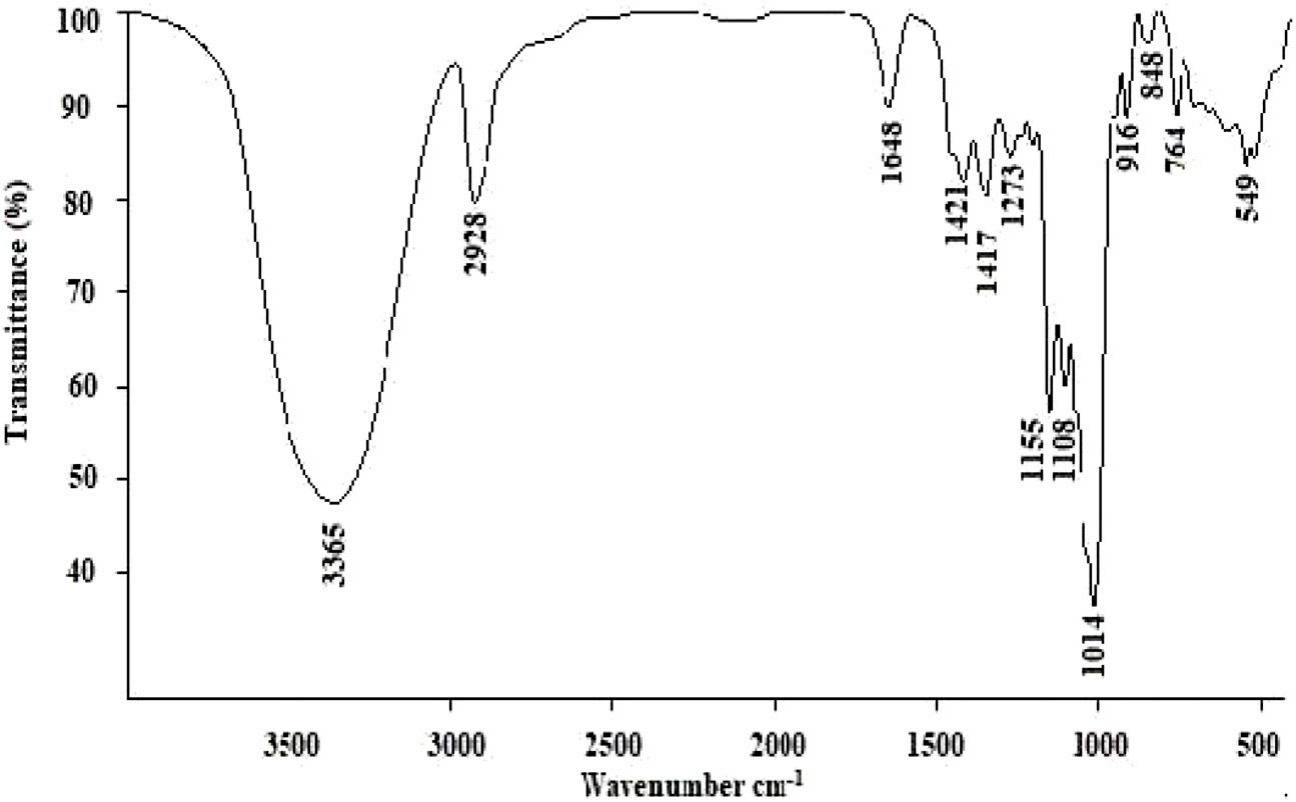
FT-IR spectral analysis of LP4.

As shown in Figure 2, the band at 3,365 cm^−1^ represented -OH stretching, and the band at 2,928 cm^−1^ was -CH stretching and bending vibrations (Xu et al., 2016). The absorption bands at 1,648 cm^−1^ were identified as the stretching vibrations of -C=O or -CHO. In addition, the band at 1,421 cm^−1^ indicated the presence of uronic acids (Wang et al., 2014). The band at 1,155 cm^−1^ was attributed to the stretching vibration of C-O (Yi et al., 2012). The absorption bands at 1,155 cm^−1^ and 1,014 cm^−1^ indicated a furanose form of sugar, which confirmed by the furanose characteristic band at 848 cm^−1^ (Ying et al., 2011).

### NMR Analysis

**FIGURE 3:**
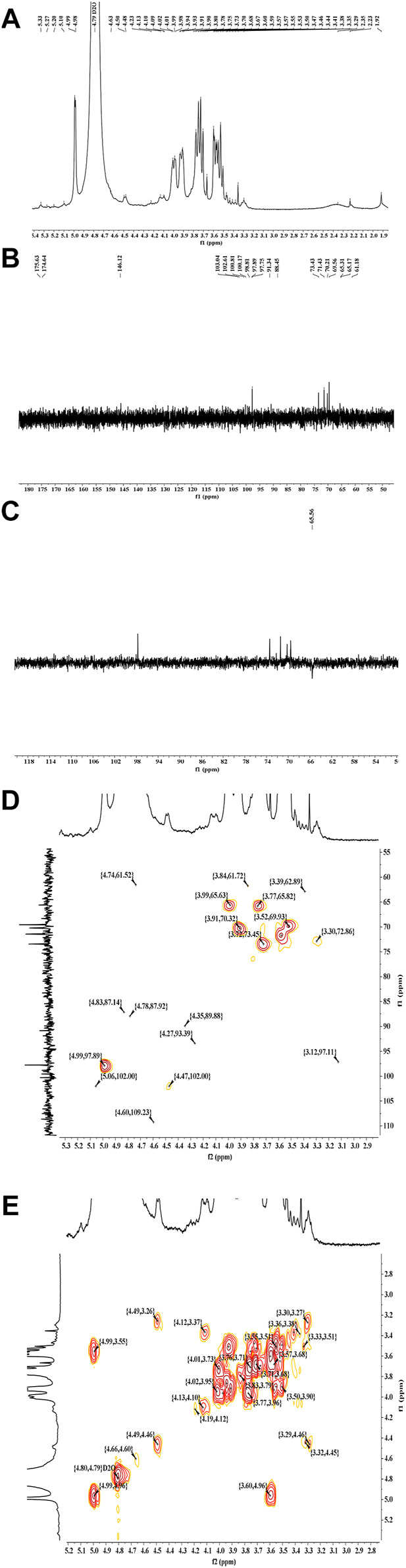
The NMR spectra analysis of LP4. ^1^H NMR **(A)**,^13^C NMR **(B)**, DEPT **(C)**, HSQC **(D)**, and COSY **(E)** spectra were recorded using an AV-400 MHz spectrometer at 30°C.

Figure 3 shows the ^1^H (A), ^13^C (B), DEPT (C), HSQC (D) and COSY (E) spectrums of LP4. The chemical shifts at *δ* 3.20-δ 5.50 ppm in ^1^H NMR spectrum were the signals of polysaccharide and the signals in *δ* 4.4–5.8 ppm region represented the anomer of sugar residue (Song et al., 2019; Tian et al., 2020). Ordinarily, the signals at *δ* 4.4–5.0 ppm represent *β*-configuration and *α*-configuration distributed in *δ* 5.0–5.8 ppm (Liu et al., 2018). The HSQC spectrum (Figure 3D) showed the main anomeric signals with H1/C1 values at *δ* 4.98/δ 97.89. The proton signals of H2, H3, H4, H5, and H6 were acquired from the cross-peaks in 1H-1H COSY spectrum (Figure 3E) at *δ* 3.30, 3.52, 3.77, 3.91, and 3.99 ppm, respectively. The correlations between the proton and carbon signals, H2 (δ 3.30)/C2 (δ 72.86), H3 (δ 3.52)/C3 (δ 69.93), H4 (δ 3.77)/C4 (δ 65.82), H5 (δ 3.91)/C5 (δ 70.32) and H6 (δ3.99)/C6 (δ 65.63), could be assigned from the HSQC spectrum (Figure 3D). Combined with previous reports (Riccio et al., 1996; Song et al., 2019), the sugar residue was identified as (1→6)-*β*-Man. Using a similar approach, the signals H1 (δ 4.99)/C1 (δ 97.75), H2 (δ 3.58)/C2 (δ 71.16), H3 (δ 3.72)/C3 (δ 72.92), H4 (δ 3.52)/C4 (δ 69.56), H5 (δ 3.91)/C5 (δ 69.79) and H6 (δ3.77)/C6 (δ 65.29) was considered to be (1→6)-*α*-glucose (Zhu et al., 2013; Luo et al., 2008; Cui et al., 2008). Moreover, the strong signal of C4 (δ 69.56 ppm, Figure 3B) suggested that the high proportion of (1→4)-linked glucose. The low signal at 4.47/102.00 ppm (H1/C1) in HSQC spectrum (Figure 3D) was attributed to (1→4)-*β*-glucose (Yu et al., 2016). Others anomer signals (δ 4.63, 5.20, 5.27 and 5.33 ppm) were hard to confirm, due to relatively low response signals. It was clear that there was a small amount of galacturonic acid and glucuronic acid present in LP4 with the ^13^C signals at *δ* 174–176 ppm (Marvelys et al., 2006; Ji et al., 2020). Combined with the results of monosaccharide composition, methylation and FT-IR, the backbone of LP4 was composed of (1→6)-*β*-Man (1→4)-*β*-Glc and (1→6)-*α*-Glc.

**FIGURE 4:**
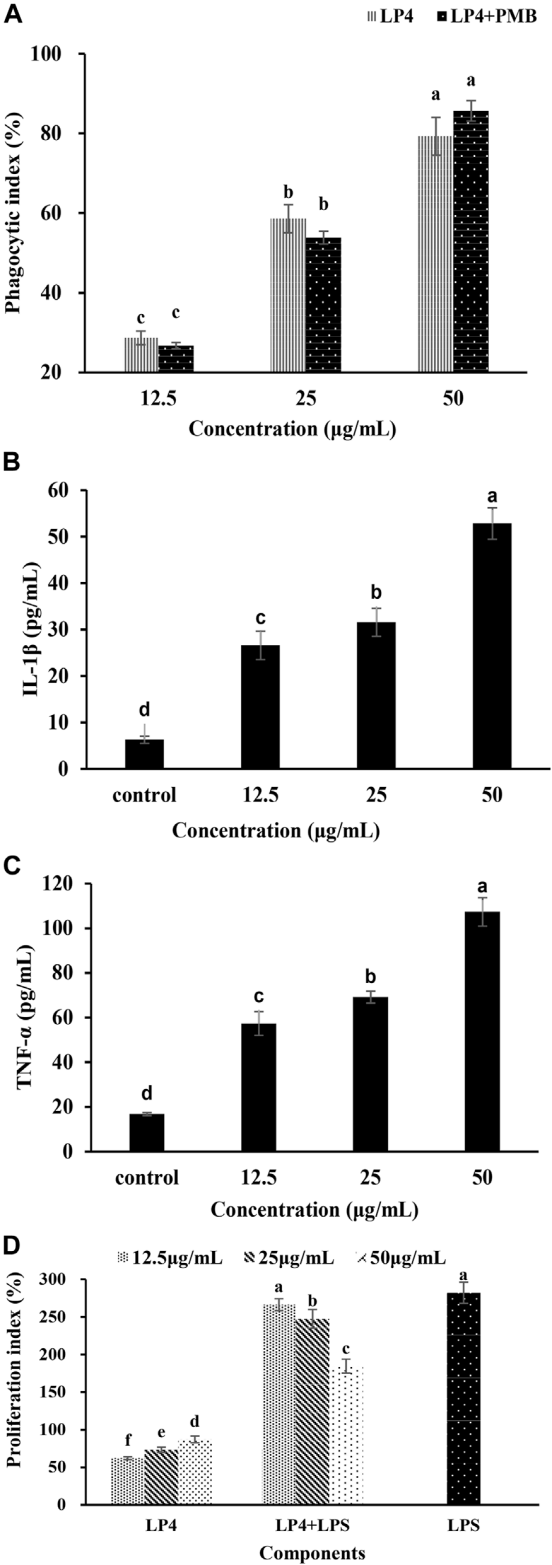
Effects of LP4 to macrophages and the proliferation of splenic lymphocyte. Note: **(A)** Effects of LP4 (12.5~50 μg/mL) on macrophages phagocytosis. **(B)** and **(C)** were the effects of LP4 on IL‐1β and TNF‐α secretion of macrophages, respectively. **(D)** Effects of LP4 on the proliferation of splenic lymphocyte. All data was expressed as means ± standard deviation (n = 6). Different superscript letters above the bar are significantly different (*p* < 0.05).

### Immunoregulatory Activity Analysis

#### Phagocytosis of Macrophages Analysis

As shown in Figure 4A, LP4 can promote the pinocytic activity of RAW264.7 cells and show a concentration-dependent at the concentration from 12.5 to 50 μg/ml. When cells were treated with 50 μg/ml LP4, the phagocytosis increased to 179.30%. Moreover, when treatment macrophages with PMB (1,000 units/mL) plus LP4 (12.5, 25 and 50 μg/ml), the phagocytosis was no significant difference with the group treated with LP4 only, which indicated that LP4 was not contaminated by LPS. These results indicated that LP4 can activate the phagocytosis of macrophages.

#### The Production of IL-1β and TNF-α Analysis

As shown in Figure 4, the production of IL-1β (Figure 4B) and TNF-α (Figure 4C) were increased obviously and showed a concentration-dependent relationship, after treatment macrophages with LP4 (12.5 ~ 50 μg/ml). After treating macrophages with 50 μg/ml LP4, the IL-1β and TNF-α productions increased 8.39 times and 6.40 times, compared to the control group, respectively.

#### Proliferation of Splenic Lymphocytes Analysis

As shown in Figure 4D, LP4 significantly enhanced lymphocyte proliferation in a concentration-dependent manner (12.5 ~ 50 μg/ml, *p* < 0.05). Compared with control, 50 μg/ml LP4 treatment increased the proliferation indices of splenic lymphocytes by 87.2%. In combination with LPS, the splenic lymphocytes increased 266.4% (12.5 μg/ml LP4), 247.5% (25 μg/ml LP4) and 184.6% (50 μg/ml LP4) of the control value. The proliferation indices of the LP4 plus LPS groups were significantly higher than that of LP4 groups but lower than that of LPS group. The result might due to LP4 and LPS competing for common receptors present on the cell surface. Consistent with activation of macrophages, these results suggested that LP4 activate the immune system.

### Membrane Receptor Analysis

**FIGURE 5:**
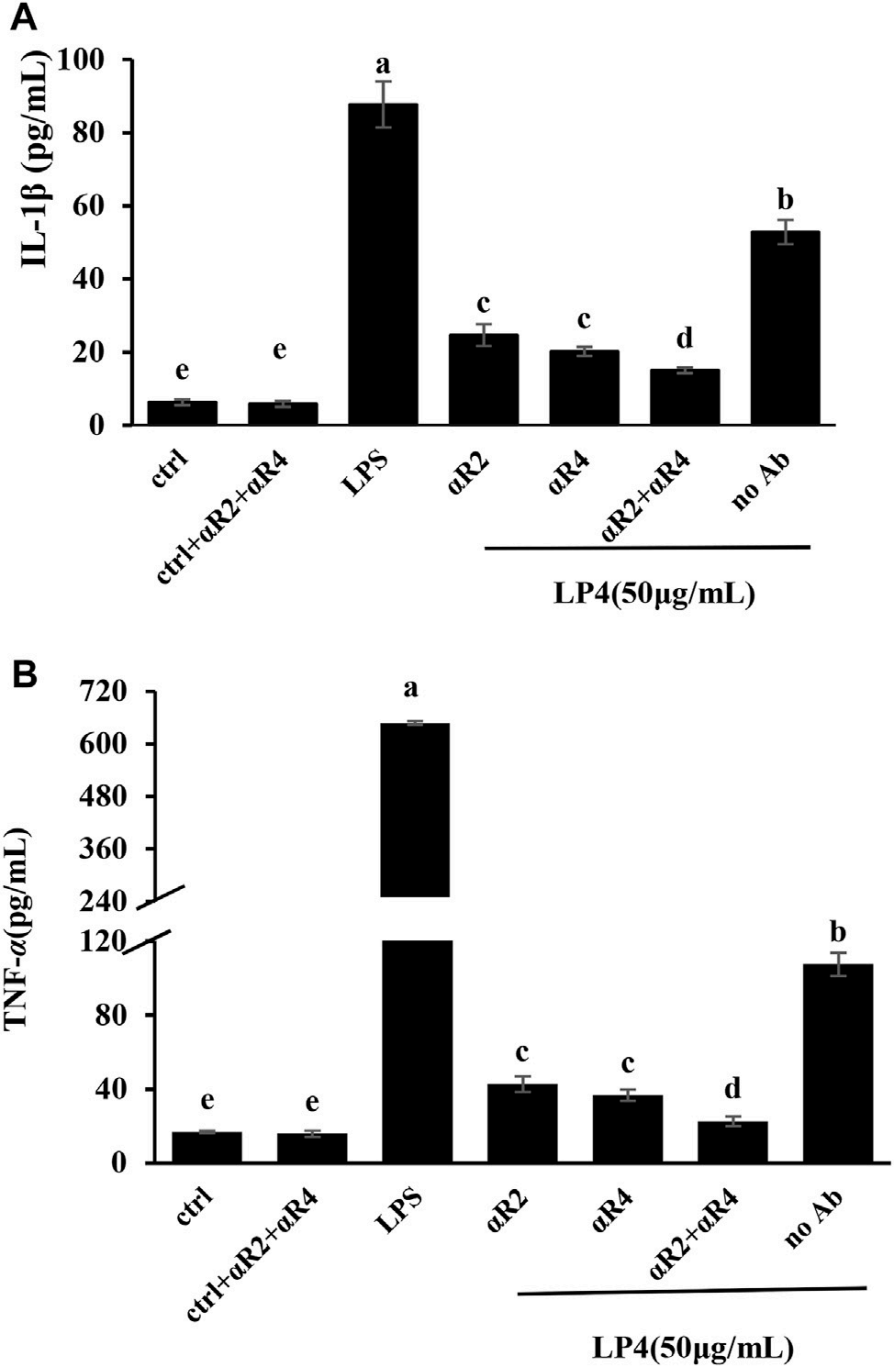
Effects of anti-TLR2 and anti-TLR4 antibodies on LP4-induced IL-1β and TNF-α secretion. RAW 264.7 cells were pre-treated with 0.1 μg/ml anti-TLR2 (aR2 group), anti−TLR4 (aR4 group), anti−TLR2 plus anti−TLR4 (aR2 + aR4 group, 0.1 μg/ml anti−TLR2 and 0.1 μg/ml anti−TLR4 antibodies) separately for 30 min before adding LP4 (50 μg/ml). The cells were pre-incubated with the medium (do not add antibodies) for 30 min and then LP4 (50 μg/ml), equal volume of medium or LPS (10 μg/ml) was added as the LP4 group (no Ab group), control group (ctrl group) and positive group (LPS group), respectively. After pre-treated with 0.1 μg/ml anti-TLR2 plus 0.1 μg/ml anti-TLR4 for 30 min, the cells were added the equal volume of medium as the antibodies control (ctrl + aR2 + aR4 group). After 24 h, IL-1β **(A)** and TNF-α **(B)** in the culture supernatants were measured by ELISA. All data was expressed as means ± standard deviation (*n* = 6). Different superscript letters above the bar are significantly different (*p* < 0.05).

To confirm the membrane receptors involved in LP4-induced macrophage activation, the macrophages were preprocessed by anti-TLR2, anti-TLR4 and anti-TLR2/4. As shown in Figure 5, contrasting with the group treated with LP4 only (No Ab group), the levels of TNF-α and IL-1β were significantly suppressed after treating with anti-TLR2, anti-TLR4 and anti-TLR2/4 (*p* < 0.05). There were no significant differences between treatment with anti-TLR2 and anti-TLR4 (*p* > 0.05). IL-1β and TNF-α were 28.34 and 21.05% after treating with anti-TLR2/4, compared with the group treated with LP4 only, respectively. Moreover, the group with anti-TLR2/TLR4 but without LP4 (ctrl + aTLR2 + aTLR4 group) did not shown difference from the control group (ctrl group). These results suggest that LP4 induce macrophage activation mainly *via* TLR2 and TLR4 receptor.

### Regulatory Pathways Analysis

**FIGURE 6:**
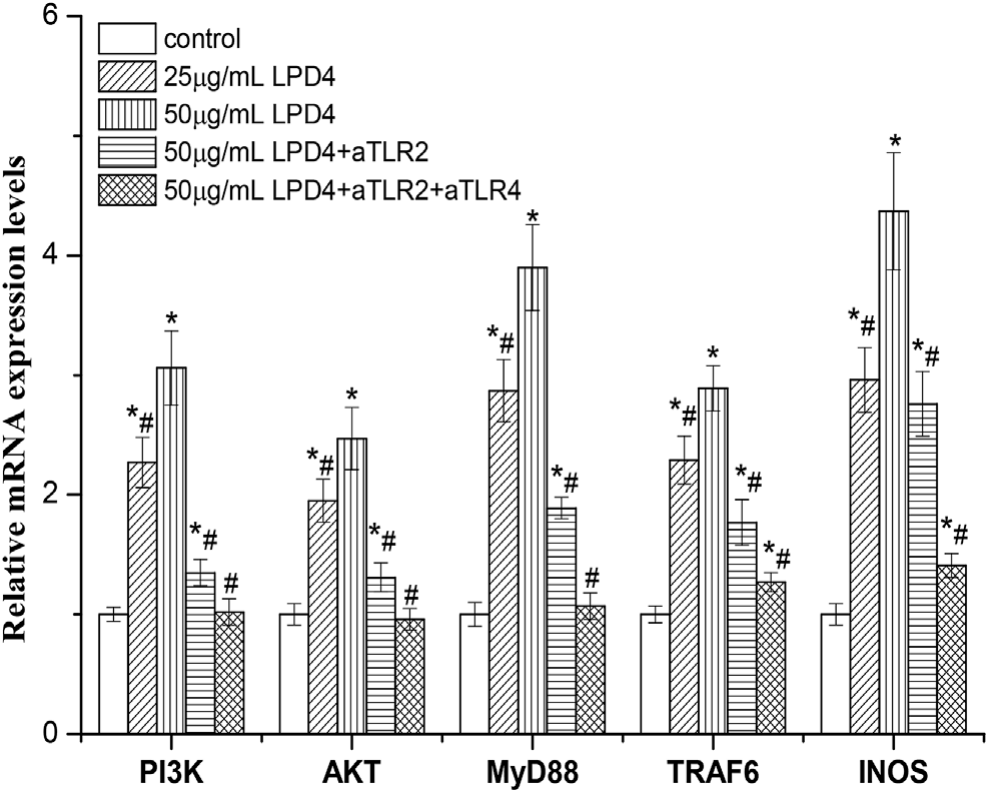
Effect of LP4 on the gene expressions of PI3K, AKT, MyD88, TRAF6, and INOS of macrophage. After RAW 264.7 cells were pre-treated with or without anti-TLR2 (0.1 μg/ml) or anti-TLR2 plus anti-TLR4 (0.1 μg/ml anti-TLR2 and 0.1 μg/ml anti-TLR4) antibodies, the cells were incubated with LP4 (25 μg/ml and 50 μg/ml) for 4 h (*n* = 4). The mRNA expression levels detected by RT-PCR were relative to that of the reference gene, *β*-actin. *p < 0.05 compared with the control group; #*p* < 0.05 compared with the 50 μg/ml LP4 group.

As shown in Figure 6, LP4 (12.5 ∼ 50 μg/ml) up-regulated the expression of INOS, PI3K, AKT, MyD88, and TRAF6 mRNA of macrophage, in a concentration-dependent manner. When treated with 50 μg/ml LP4, the expression levels of INOS, PI3K, AKT, MyD88, and TRAF6 were 4.37 ± 0.28, 3.06 ± 0.19, 2.47 ± 017, 3.90 ± 0.24, and 2.89 ± 0.16 times as many as the control group, respectively. Moreover, the expression of these genes was obviously decreased when treated with anti-TLR2 and anti-TLR2/4. The expression levels of INOS, PI3K, AKT, MyD88, and TRAF6 were reduced to 2.76 ± 0.20, 1.35 ± 0.11, 1.31 ± 0.09, 1.89 ± 0.14, and 1.77 ± 0.09 times (compare with control group) after treatment with anti-TLR2. Meanwhile, the expression levels of these genes decreased to 1.41 ± 0.11, 1.02 ± 0.06, 0.96 ± 0.08, 1.07 ± 0.05, and 1.20 ± 0.07 of the control group after treatment with anti-TLR2/4, respectively. These results indicate that TLR2 and TLR4-mediated PI3K/AKT and MyD88/TRAF6 pathways involved in the activation of LP4 to macrophages.

## Discussion

The characterization of main active components, active mechanism, and structure–function relationship are the basis of the function and application of natural products. In recent years, several active polysaccharides were used as therapeutic agent for treatment disease (Yu et al., 2018). However, the application of many polysaccharides from natural sources was limited due to the complex structure and unclear structure-activity relationship (Harding et al., 2017). In the present study, a novel active polysaccharide of longan (LP4), which main linkages were (1→6)-*β*-Man (1→4)-*β*-Glc and (1→6)-*α*-Glc with average molecular weight of 6.31 × 10^4^ g/mol, was characterized and its immunomodulation activities were evaluated. The structure and macrophage-activating mechanism of LP4 are dramatically different from longan polysaccharides in previous reports (Yi et al., 2011; Meng et al., 2014; Rong et al., 2019). The comparative analysis of structures and activities may help reveal the structure–function relationships and uncover the multiple molecular mechanism of activating macrophages of longan polysaccharides. Previous studies demonstrated that the bioactivity of polysaccharide was closely associated with their structural features (Ferreira et al., 2015). The (1→4)-Glc (1→6)-Glc, and (1→6)-Man were found to be the main backbone for many polysaccharides with immunomodulatory activity (Ferreira et al., 2015; Yamabhai et al., 2016; Yan et al., 2018), including longan polysaccharides (Rong et al., 2019; Gan et al., 2021). The linkage characteristics of (1→4)-Glc (1→6)-Glc and (1→6)-Man may contribute to immunoregulatory activity of LP4. In previous studies, it has been proposed that polysaccharides with considerable activities normally have high molecular weight and acetyl or sulfuric acid functional groups (Ferreira et al., 2015; Rong et al., 2019). However, the current study results show that the high molecular weight and acetyl or sulfuric acid functional groups are not the necessarily structural features of longan polysaccharide with strong immunoregulatory activity. LP4 has a relatively lower molecular weight (6.31 × 10^4^ g/mol) and no acetyl or sulfuric acid groups, while it showed strong immunoregulatory activity.

**FIGURE 7:**
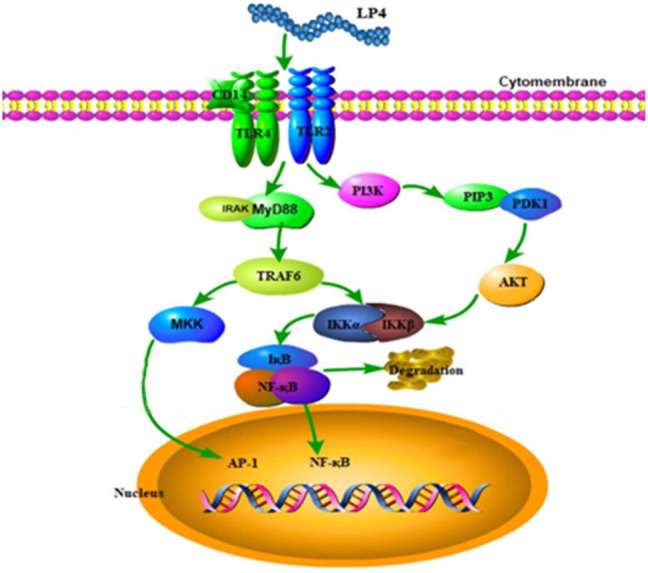
Possible molecular mechanism of LP4 activating the macrophages.

Polysaccharide mediated immune regulation is a complex process. The triggering of cellular immune response by polysaccharides mainly depends on pattern recognition receptors on immune cells (Leung et al., 2006). Toll-like receptors play an important role in polysaccharides induced macrophage activation. Meanwhile, the PI3K/AKT and MyD88 pathways play a key role in the TLR2-and TLR4-mediated activation of macrophages, respectively (Feng et al., 2016; Vergadi et al., 2017). Previous studies showed that several polysaccharides from natural sources activated macrophage through TLR2 and TLR4-mediated MAPK/NF-kappa B (Wang et al., 2018a; Yang et al., 2019) and transcriptional activities of activator protein-1 (Shen et al., 2017). In this study, we found that LP4 induced activation of macrophages *via* TLR2 and TLR4 mediated with PI3K/AKT and MyD88/TRAF6 pathways. In our early study, the dried longan pulp polysaccharide LPD2 with (1→4)-*β*-Glc and (1→6)-*β*-Man glycosidic linkages was not shown to activate macrophages *via* TLR2-mediated PI3K/AKT pathway (Rong et al., 2019). The difference in the structure of the two polysaccharides may lead to the difference in the signal pathway of macrophages activation. There are differences in the mechanism of activating macrophages by longan polysaccharide with different structures. In the present study, the expression of INOS and TRAF6 induced by LP4 was not completely suppressed by anti-TLR2/4. These results suggested that there may be other signal pathways or receptors involved in LP4-mediated macrophage activation. In summary, LP4 induced the activation of macrophage mainly *via* the TLR2 and TLR4-induced PI3K/AKT and MyD88/TRAF6 pathways (Figure 7).

## Conclusion

In this work, a novel immunoreactive polysaccharide with average molecular weight of 6.31 × 10^4^ g/mol was isolated from fresh longan. It was composed of mannose, glucose, glucuronic acid, galactose, xylose, arabinose, galacturonic acid, fucose, and rhamnose in a molar percentage of 36:31:10:7:4:4:3:2:2 and mainly linked by (1→6)-*β*-Man, (1→4)-*β*-Glc, and (1→6)-*α*-Glc. LP4 can promote the proliferation of lymphocytes and enhanced the phagocytosis of macrophage. Moreover, LP4 induced the activation of macrophage mainly *via* the TLR2 and TLR4-mediated PI3K/AKT and MyD88/TRAF6 pathways.

## Data Availability

The raw data supporting the conclusions of this article will be made available by the authors, without undue reservation.
