# Supplementary material for: A Novel Polysaccharide Isolated From Fresh Longan (Dimocarpus longan Lour.) Activates Macrophage via TLR2/4-Mediated PI3/AKT and MyD88/TRAF6 Pathways
Source: Front Pharmacol. 2021 Dec 21;12:786127. doi: 10.3389/fphar.2021.786127 (PMC8724522; doi:10.3389/fphar.2021.786127)

**Table S1 Primer sequences used in the present study.**

| **Genes** | **Primer sequences** |
| --- | --- |
| **PI3K** | Foreword GGTGGTCACGTTGCTAAGC  Reverse CGCAGGTGCCAATCATTCTTAT |
| **AKT** | Foreword GTCCTGCAGAACTCTAGGCATC  Reverse CACGAGACAGGTGGAAGAAGAG |
| **MyD88** | Foreword CTAATTGAGAAAAGGTGTCGCCGC  Reverse AATCTGGCTCCGCATCAGTCTC |
| **TRAF6** | Foreword GTACGATCGGGTTGTGTGTG  Reverse ACACCCCAGCAGCTAAGAAC |
| **iNOS** | Foreword ACATCGACCCGTCCACAGTAT |
|  | Reverse CAGAGGGGTAGGCTTGTCTC |
| **ß-actin** | Foreword GGGTCAGAAGGACTCCTATG  Reverse GTAACAATGCCATGTTCAAT |

**Figure 1S. The DEAE eluting profiles of LP4.**


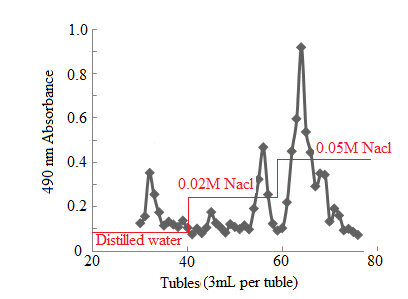


**Figure 2S. GC analysis the monosaccharide composition of LP4.**

Note: 1: Rhamnose, 2: Fucose, 3: Arabinose, 4: Xylose, 5: Mannose, 6: Glucose, 7: Galactose, 8: Glucuronic acid, 9: Galacturonic acid


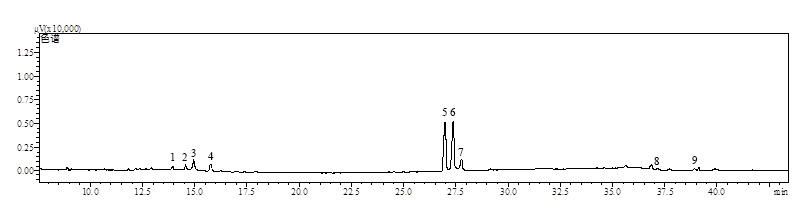

Supplement: Supplementary file 1 [file DataSheet1.docx]
